# Evaluation of spice and herb as phyto-derived selective modulators of human retinaldehyde dehydrogenases using a simple *in vitro* method

**DOI:** 10.1042/BSR20210491

**Published:** 2021-05-20

**Authors:** Thi Bao Chau Bui, Shohei Nosaki, Mito Kokawa, Yuqun Xu, Yutaka Kitamura, Masaru Tanokura, Satoshi Hachimura, Takuya Miyakawa

**Affiliations:** 1 Department of Applied Biological Chemistry, Graduate School of Agricultural and Life Sciences, The University of Tokyo, 1-1-1 Yayoi, Bunkyo-ku, Tokyo 113-8657, Japan; 2 Research Center for Food Safety, Graduate School of Agricultural and Life Sciences, The University of Tokyo, 1-1-1 Yayoi, Bunkyo-ku, Tokyo 113-8657, Japan; 3 Graduate School of Science and Technology, University of Tsukuba, 1-1-1 Tennodai, Tsukuba, Ibaraki 305-8572, Japan; 4 Faculty of Life and Environmental Sciences, University of Tsukuba, 1-1-1 Tennodai, Tsukuba, Ibaraki 305-8572, Japan

**Keywords:** Fluorescence activity assay, Protein preparation, Retinal, Retinaldehyde dehydrogenase, Selective modulator

## Abstract

Selective modulation of retinaldehyde dehydrogenases (RALDHs)—the main aldehyde dehydrogenase (ALDH) enzymes converting retinal into retinoic acid (RA), is very important not only in the RA signaling pathway but also for the potential regulatory effects on RALDH isozyme-specific processes and RALDH-related cancers. However, very few selective modulators for RALDHs have been identified, partly due to variable overexpression protocols of RALDHs and insensitive activity assay that needs to be addressed. In the present study, deletion of the N-terminal disordered regions is found to enable simple preparation of all RALDHs and their closest paralog ALDH2 using a single protocol. Fluorescence-based activity assay was employed for enzymatic activity investigation and screening for RALDH-specific modulators from extracts of various spices and herbs that are well-known for containing many phyto-derived anti-cancer constituents. Under the established conditions, spice and herb extracts exhibited differential regulatory effects on RALDHs/ALDH2 with several extracts showing potential selective inhibition of the activity of RALDHs. In addition, the presence of magnesium ions was shown to significantly increase the activity for the natural substrate retinal of RALDH3 but not the others, while His-tag cleavage considerably increased the activity of ALDH2 for the non-specific substrate retinal. Altogether we propose a readily reproducible workflow to find selective modulators for RALDHs and suggest potential sources of selective modulators from spices and herbs.

## Introduction

**Figure 1:**
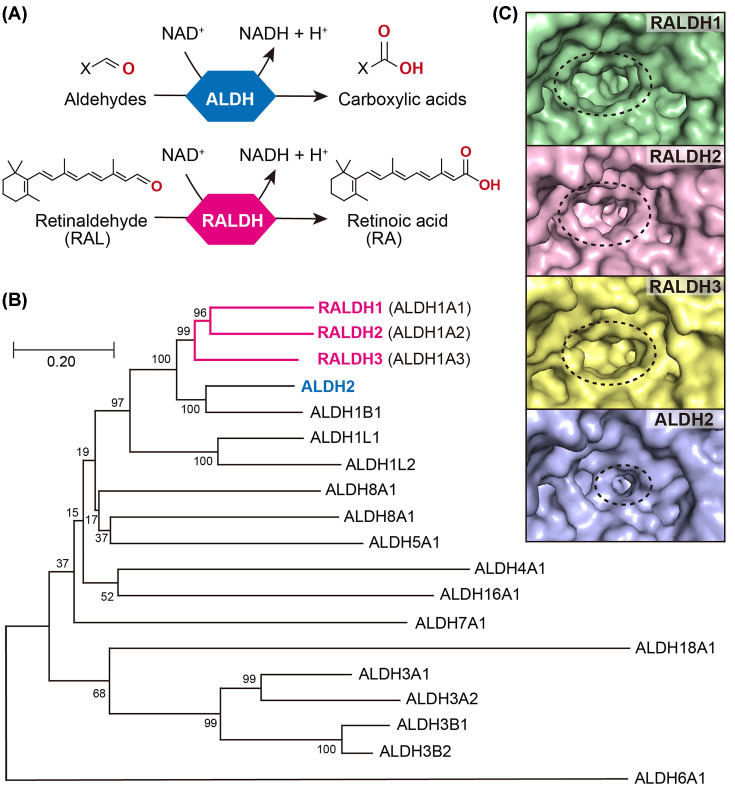
Features of human RALDHs and ALDH2 (**A**) Catalysis of acetaldehyde to acetic acid by ALDH/RALDH family proteins. (**B**) Phylogenetic tree of 19 human *ALDH* genes encoding for corresponding members of ALDH superfamily. The percentages of replicate trees in which the associated taxa clustered together in the bootstrap test (500 replicates) are shown next to the branches. Branch lengths are in the same units as those of the evolutionary distances. (**C**) The substrate tunnel entrances of RALDHs and ALDH2 shown as surface models and are surrounded by a black dotted line. Surface representation of RALDH1-3 and ALDH2 were created based on Protein Data Bank (PDB) code 4WB9, 6ALJ, 5FHZ (chain D) and 1O01, respectively, and depicted using the molecular graphics system PyMOL (Ver. 2.4, Schrodinger, LLC).

In living organisms, metabolism pathways are stricly maintained not only to provide sufficient energy and nutrients for survival and activity but also to eliminate harmful substances. Aldehyde dehydrogenases (ALDHs) comprise a superfamily of enzymes that participate in such pathways, catalyzing the oxidation of toxic endogenous and exogenous aldehydes into corresponding carboxylic acids in the presence of the nicotinamide adenine dinucleotide (NAD^+^) cofactor (Figure 1A). In the human genome, 19 *ALDH* genes have been discovered, encoding for 19 corresponding ALDH proteins with conserved catalytic residues (Figure 1B) [1]. To date, ALDH2 has been one of the most well-studied human ALDHs because of its crucial role for the alcohol metabolism in the liver, catalyzing the oxidation of acetaldehyde to acetic acid [2]. However, focus has also recently been aimed at the ALDH1A subfamily owing to their critical and differential roles for the retinoic acid (RA) signaling pathway in mainly growth and development, as well as the immune system and cancer regulation [3]. There are three isozymes [1–3] in ALDH1A subfamily whose natural specific substrate is retinaldehyde (or retinal, RAL) [4]. Because ALDH1A1–3 are the main ALDHs that specifically convert RAL into RA—the major functional metabolite of RA signaling, they are commonly regarded as retinaldehyde dehydrogenases (RALDHs) (Figure 1A,B). Although human RALDHs and ALDH2 share high similarity (over 70%, Figure 1B and Supplementary Figure S1), structural studies have revealed that the substrate tunnel entrances of human RALDH1–3 are larger than that of ALDH2 and adopt distinct conformations for selectively accommodating the bulky RAL molecule [5] (Figure 1C).

Despite sharing high similarity in amino acid sequences (over 80%) and three-dimensional structures (root-mean-square deviation (RMSD) < 0.8 Å between each protomer) (Supplementary Figures S1 and S2), RALDHs not only participate in different processes in different tissues, but are also regulated diversely, especially in cancer cells [5,6]. Many studies have revealed that RALDHs, especially RALDH1 and RALDH3, are strongly linked to cancer stem-like cells (CSCs) with increasing evidence on up-regulated expression and heightened activity in correlation with tumor aggressiveness, treatment resistance and metastasis [7]. Pan-inhibitors against RALDHs, such as compound 673A (4-(1,3-dihydro-2*H*-isoindol-2-yl)benzaldehyde) and DIMATE (4-dimethylamino-4-methyl-pent-2-ynthioic acid *S*-methyl ester, or dimethyl ampal thiolester), have been shown to be effective in targeting CSCs *in vitro* such as ovarian and leukemia CSCs [8–10]. However, RALDH inhibitors can also target and inhibit other ALDH family enzymes, including the closest paralog ALDH2, resulting in off-target effects such as blurred vision, nausea and flushing [11–13]. For that reason, recent attempts have also yielded a limited number of potential inhibitors with high specificity by screening for analogs of available inhibitors such as CM026, CM037, NCT-505 (RALDH1-specific), 15g and 15l (RALDH3-specific) or even by synthesis such as dichloro-all-*trans*-retinone (RALDH2-specific) [14–16]. However, the fact that these compounds are synthetic and/or not from natural sources raises the question of their applicability as well as possible adverse effects on human health upon long-term usage.

On the other hand, practices using natural phytochemicals are increasingly reported for medicinal purposes, including tumor-suppressing therapies [17]. Several RALDHs/ALDHs inhibitors that may have the potential in treatments of RALDHs/ALDHs-related diseases are in fact phytochemicals such as citral (from citrus fruits) and daidzin (from Japanese kudzu or soybean leaves) [18,19]. However, most phyto-derived RALDHs/ALDHs inhibitors lack specificity and may cause undesirable suppression of other essential RALDHs/ALDHs functions. While spices and herbs are the reservoirs of a plethora of phytochemicals with formidable effects on human health and lower risks of side effects, no studies have been conducted on the effects of whole spice and herb extracts on RALDHs. Conventional inhibitors are mostly non-natural, poorly tolerated and have low specificity, whereas working mechanisms of ALDHs/RALDHs are highly isozyme-specific especially in cancer cells. Therefore, it is essential to find alternative selective inhibitors that are preferably from natural sources, among which spices and herbs are potential candidates. Screening for selective effects of various extracts or compounds on RALDHs requires all isozymes of RALDH as well as their close paralogs such as ALDH2 heterologously expressed in large quantities and highly purified for comparison. Nonetheless, protocols for preparation of RALDHs and ALDH2 vary from one study to another in terms of expression system and purification method, which complicates the overall process of preparing enzymes for screening studies [20–23].

In the present study, we developed a simplified and common method to produce RALDHs and ALDH2 proteins of high purity using only one expression system and purification protocol. We also established an indirect but sensitive enzyme activity assay, aiming to screen for potential modulating effects on RALDHs with high selectivity *in vitro*. Under the conditions established, it was possible to monitor RALDHs activity with compound mixtures extracted from spices and herbs, serving as a basis for identification and characterization of natural-derived selective modulators applied in RA-signaling regulation and cancer therapies. Furthermore, with 22 spice and herb extracts showing different patterns of regulating RALDHs and ALDH2, our results show that some spices and herbs are potential sources of effective and selective modulators of RALDHs. Altogether, our protocol and obtained results may greatly facilitate not only cognitive but also practical studies on RALDHs and ALDH2.

## Results and discussion

### Simple preparation of recombinant RALDHs/ALDH2 proteins

**Figure 2:**
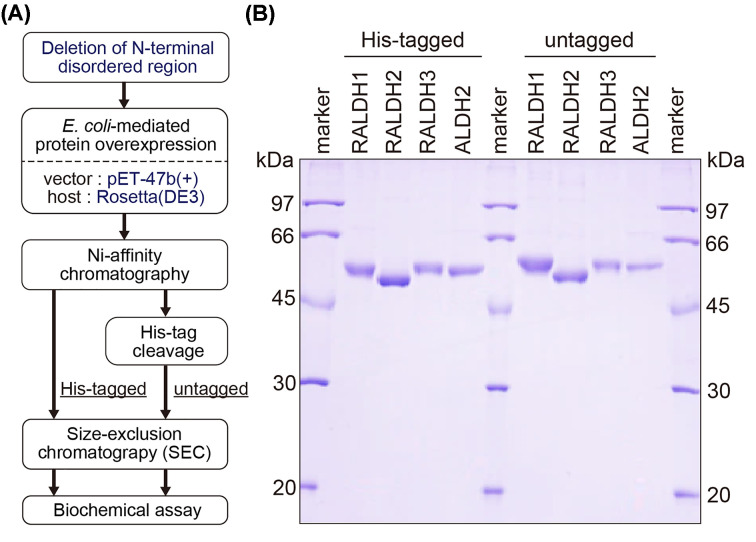
Preparation of His-tagged and untagged RALDHs and ALDH2 proteins (**A**) Schematic flowchart of the expression and purification of RALDHs/ALDH2. (**B**) Coomassie-stained SDS/PAGE analysis of the purified His-tagged and untagged RALDHs/ALDH2.

For determination of activity in biochemical assays, we attempted to obtain *Escherichia coli*-mediated recombinant RALDHs/ALDH2 proteins of high purity by a simplified and universal method as the first step towards the screening for potential modulators derived from spices and herbs (Figure 2A). In the present study, the first 8–25 N-terminal residues of RALDHs/ALDH2 were deleted due to their predicted disorder (Supplementary Figure S1). The N-terminus deleted RALDHs/ALDH2 were successfully expressed into the soluble fraction using the pET-47b(+) vector for N-terminal His-tag fusion and *E. coli* host strain Rosetta(DE3). His-tagged RALDHs/ALDH2 were applied to affinity chromatography using a nickel chelating resin (Ni-affinity chromatography) with or without His-tag cleavage by the human rhinovirus 3C (HRV 3C) protease. Both His-tagged and untagged proteins were further purified by size-exclusion chromatography (SEC). After collecting the non-aggregated fractions, we confirmed to acquire each protein at high purity by sodium dodecyl sulfate/polyacrylamide gel electrophoresis (SDS/PAGE) (Figure 2B).

Despite high similarity in amino acid sequence, there is no common expression system for successful preparation of three RALDH isozymes as well as ALDH2. While in many studies full-length coding sequences were successfully used to express RALDH proteins [20,21,23–28], the use of truncated or modified stretches was also reported [5]. ALDH2 is a mitochondrial protein and thus is expected to carry an N-terminal leader sequence which is cleaved to form mature protein [29]. Meanwhile, RALDHs are cytosolic proteins, and the roles of their disordered N-terminal regions remain controversial [29,30]. However, disordered regions in heterologous protein expression in *E. coli* may interfere with the protein assembly and folding due to incompatibility with *E. coli* chaperones [31]. In fact, an attempt to express full-length RALDH2 as a representative for RALDHs resulted in a majority of proteins remaining in the insoluble fraction, unlike the N-terminus deleted RALDH2 (Supplementary Figure S3). This could be due to the accumulation of the misfolded RALDH2. Consequently, deletion of this first N-terminal stretch facilitated *E. coli*-mediated overexpression of RALDHs/ALDH2 and may affect the expression of other ALDH superfamily members in a similar way. In addition, all three investigated RALDH isozymes together with ALDH2 were successfully prepared using the same common vector–host strain (pET-47b(+)-Rosetta(DE3)), followed by efficient two-step chromatography purification. Given that the combination of vectors, host cells as well as purification steps differ completely from one RALDH/ALDH isozyme to another in previous studies [23,32], our protocol may be highly useful for the simultaneous investigation that requires overexpression and purification of all RALDHs/ALDH2.

### Magnesium ion and N-terminal His-tag have differential effects on the RAL dehydrogenation activity of RALDHs and ALDH2

**Figure 3:**
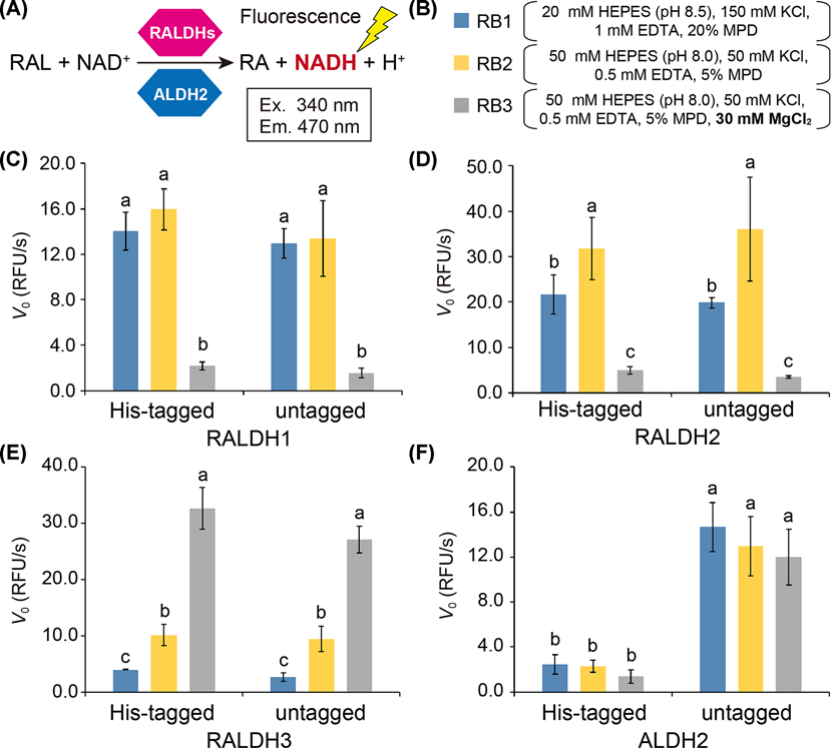
Effects of Mg^2+^ and His-tag on the activity of RALDHs and ALDH2 (**A**) Catalysis of RAL to RA by RALDHs/ALDH2, coupled with the generation of NADH from the cofactor NAD^+^, emitting fluorescence signals. (**B**) Three different buffer composition without Mg^2+^ (RB1, 2) and with Mg^2+^ (RB3). HEPES, 4-(2-hydroxyethyl)-1-piperazineethanesulfonic acid; EDTA, ethylenediaminetetraacetic acid; and MPD, 2-methyl-2,4-pentanediol. (**C**–**F**) Effects of buffers RB1–3 and the absence or presence of His-tag on initial velocity (*V*_0_) of reactions for RALDH1 (C), RALDH2 (D), RALDH3 (E) and ALDH2 (F). Blue, yellow and gray colored graphs represent RB1–3 buffers, respectively. Error bars represent standard deviation of mean at 95% confidence (*n*=3). Statistical analysis including analysis of variance followed by Tukey’s post-hoc test was performed separately for each protein under two tag conditions (His-tagged, untagged) and three buffer conditions. Means that do not share a letter are significantly different (*P*<0.05).

To optimize the biochemical assay conditions for RALDHs/ALDH2 activity measurement, we investigated the effects of buffer composition and N-terminal His-tag on the enzymatic activity of RALDHs/ALDH2. Reactions were conducted with the natural all-*trans*-retinal (atRAL) substrate and each enzyme activity was measured by monitoring the NADH-derived fluorescence (Figure 3A). Three kinds of buffer were selected, including two compositions used in previous studies of RALDHs (reaction buffer 1 (RB1) and 3 (RB3)) as the representatives of those without and with Mg^2+^ respectively, as well as one composition for comparison (RB2, modified from RB3 by omitting Mg^2+^) (Figure 3B). Under the current experimental conditions with these buffers for RALDHs, we observed the activity for atRAL substrate of not only RALDHs but also ALDH2 (Figure 3C–F). The absence or presence of Mg^2+^ showed differential effects for RALDH isozymes and ALDH2. At 30 mM Mg^2+^ (RB3), significant inhibition of activity was shown for RALDH1 and RALDH2 regardless of His-tagged or untagged, with initial velocity *V*_0_ for both enzymes lowered by up to 90% compared to that in the absence of Mg^2+^ (RB1 and RB2) (Figure 3C,D). No significant effect was observed for ALDH2, despite a tendency of reaction being slightly slowed down by Mg^2+^ (Figure 3F). On the contrary, Mg^2+^ caused RALDH3 to exhibit a remarkable burst in the activity for atRAL substrate (Figure 3E). In addition, an upsurge in RALDH3 activity was observed when Mg^2+^ concentration increased from 0.01 to 1 mM, followed by a more gradual rise in the range of 1 to 10 mM (Supplementary Figure S4).

Stimulation effect of Mg^2+^ on RALDH3 was demonstrated for its natural substrate atRAL for the first time, with comparable tendency to that carried out with the alternative substrate hexanal as recently reported [27]. While previous studies showed that Mg^2+^ strongly inhibited RALDH1 in a dose-dependent manner [33,34], inhibitory effect was observed for RALDH2 and ALDH2 at high concentrations [28,35]. The regulation of RALDHs/ALDH2 by metal ions have been suggested to be due to either the protein structure solely [33,34] or the reaction mechanism (different rate-limiting step between RALDH1/2 and RALDH3/ALDH2) [35]. Although our results with the natural substrate RAL stay more consistent with the rate-limiting step theory, molecular and structural bases underlying the metal ions-dependent regulation are still under investigation.

ALDH2 isolated from biological sources have been reported to demonstrate very weak or almost no activity for retinal substrates in previous studies [36]. However, it is noteworthy that our results show the weak activity of His-tagged ALDH2 and adequate activity of untagged ALDH2 for atRAL. This suggests that in suitable buffers and conditions, untagged ALDH2 but not His-tagged ALDH2 can metabolize atRAL to a certain extent and the activity can be observed via NADH-derived fluorescence instead of absorbance. While the presence of His-tag was shown to substantially hinder the activity of ALDH2, no effect was observed for RALDHs (Figure 3C–E). Despite being inevitable in recombinant protein overexpression as a purification tool [30], terminal tags have been shown to have various effects on the constitution, pH and stability of recombinant proteins [37], and probably on RALDHs/ALDH2 as reported in the present study. Moreover, our data indicate the possibility that N-terminal His-tagged RALDHs, but not ALDH2, are suitable for other biochemical assays based on the protein immobilizing techniques with His-tag, such as surface plasmon resonance (SPR) for binding assay.

### Spice and herb extracts potentially contain selective modulators for RALDHs

**Figure 4:**
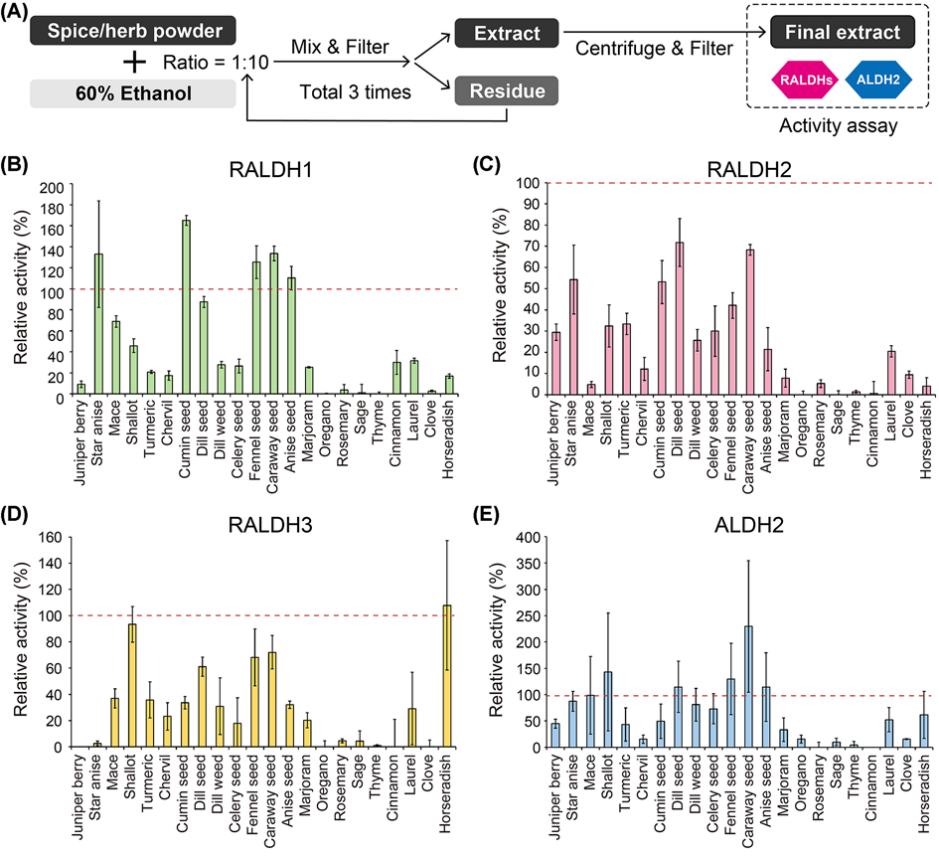
*In vitro* activity assays for RALDHs/ALDH2 using spice and herb extracts (**A**) Extraction scheme for spice and herb samples. (**B**–**E**) Relative activities of RALDH1 (B), RALDH2 (C), RALDH3 (D) and ALDH2 (E) mixed with the spice and herb extracts. Error bars represent standard deviation of mean at 95% confidence (*n*=3).

In order to find specific regulators for RALDHs, 22 ethanolic extracts of different spices and herbs were prepared and used for *in vitro* RALDHs/ALDH2 activity assays with the natural substrate atRAL of RALDHs (Figure 4A). In this experiment, untagged RALDHs/ALDH2 were used because ALDH2 activity was higher than those with His-tag (Figure 3F). For RALDH1/2 and ALDH2 inhibitory assays, RB1 was chosen over RB2 because of lower degree of instability in activity although both buffers did not contain Mg^2+^ (Figure 3C,D). To maximize enzyme activity, RALDH3 was subjected to inhibitory assays in the presence of Mg^2+^ (RB3) (Figure 3E). Interestingly, most extracts showed differential effects, either activity-increasing or -decreasing, on the tested enzymes (Figure 4B–E). A total of 15, 18 and 17 out of 22 of the tested extracts showed considerable inhibitory effects (less than 50% activity remained) on RALDH1, RALDH2 and RALDH3 respectively (Figure 4B–D), while this figure was only 12 out of 22 of all extracts for ALDH2 (Figure 4E). Several extracts showed selective inhibitory effects on two or three out of four tested enzymes, such as mace, fennel seed and anise seed on RALDH2/3, dill weed and celery seed on RALDH1/2/3, and horseradish on RALDH1/2. Remarkably, in the presence of star anise extract, RALDH3 was almost completely inhibited with the remaining relative activity of only 2.6%, while RALDH1 was in fact stimulated and RALDH2/ALDH2 still remained 54.3 and 87.3% active, respectively (Table 1). Star anise, cumin seed and caraway seed were shown to accelerate the reaction catalyzed by RALDH1, resulting in a significant increase in relative activity by up to 133, 165 and 133%, respectively.

**Table 1:** Summary of relative activities of RALDH1–3 and ALDH2 mixed with the spice and herb extracts

No.	Extract of spice/herb	Relative activity to control (%) (mean ± SD)			
		RALDH1	RALDH2	RALDH3	ALDH2
1	Juniper berry	9.2 ± 3.2	29.5 ± 3.9	N/A	44.9 ± 8.6
2	Star anise	133.1 ± 50.7	54.3 ± 16.2	2.7 ± 1.9	87.3 ± 19.1
3	Mace	69.0 ± 5.5	4.8 ± 1.4	36.8 ± 7.3	98.7 ± 74.0
4	Shallot	45.8 ± 6.5	32.4 ± 9.9	93.3 ± 13.6	143.2 ± 112.0
5	Turmeric	21.0 ± 1.5	33.4 ± 5.1	35.8 ± 13.6	43.4 ± 31.4
6	Chervil	17.3 ± 4.4	12.1 ± 5.5	23.2 ± 10.5	15.3 ± 8.1
7	Cumin seed	165.1 ± 4.8	53.1 ± 10.2	33.6 ± 4.8	49.7 ± 32.7
8	Dill seed	87.6 ± 5.2	71.7 ± 11.3	61.2 ± 7.3	114.8 ± 49.0
9	Dill weed	27.7 ± 3.2	25.7 ± 5.2	30.9 ± 21.5	80.9 ± 31.1
10	Celery seed	26.5 ± 6.6	29.9 ± 11.8	17.9 ± 19.4	73.0 ± 28.6
11	Fennel seed	125.3 ± 15.5	42.1 ± 6.1	68.3 ± 21.6	129.9 ± 67.7
12	Caraway seed	133.7 ± 6.9	68.3 ± 2.5	72.0 ± 12.7	229.6 ± 125.1
13	Anise seed	110.2 ± 11.1	21.4 ± 10.1	32.1 ± 2.8	114.2 ± 65.0
14	Marjoram	25.3 ± 0.6	7.8 ± 4.3	20.3 ± 5.7	33.4 ± 22.3
15	Oregano	N/A	N/A	N/A	15.9 ± 7.6
16	Rosemary	3.9 ± 5.1	5.3 ± 1.8	4.6 ± 1.5	N/A
17	Sage	1.0 ± 8.3	N/A	4.4 ± 7.9	10.0 ± 7.3
18	Thyme	N/A	1.4 ± 0.7	1.2 ± 0.5	4.34 ± 7.0
19	Cinnamon	29.9 ± 11.3	0.6 ± 5.6	N/A	N/A
20	Laurel	31.6 ± 2.7	20.4 ± 2.7	29.2 ± 27.6	52.2 ± 23.0
21	Clove	2.7 ± 0.8	9.4 ± 1.8	N/A	15.6 ± 0.7
22	Horseradish	17.0 ± 2.1	4.0 ± 4.0	107.8 ± 49.3	61.6 ± 44.5

Abbreviations: N/A, no activity; SD, standard deviation.

The search for natural modulators of ALDH family members, including RALDHs, have been intensively conducted for many decades. One of the most conventional and typical modulators is citral, which inhibits many ALDH enzymes such as ALDH2 and 3 [27,38,39]. Garlic-derived allyl sulfides can effectively inhibit yeast-derived non-specific ALDH [40], while several derivatives of phytochemicals such as coumarin-461 [41] have also been shown as potent inhibitors on RALDH1, but with weak inhibition of RALDH2/3. Nevertheless, no studies have reported the presence of naturally occurred inhibitors for RALDHs with high selectivity in spices and herbs, especially those investigated in the present study like fennel seed, anise seed or dill. On the other hand, although activators for enzymatic proteins are less heard of than inhibitors, previous studies have found compounds that activate some ALDH family enzymes, such as the small molecule *N*-(1,3-benzodioxol-5-ylmethyl)-2,6-dichlorobenzamide, also known as Alda-1 (for ALDH2), and the phyto-derived monoterpene limonene (LI) and cruciferous sulforaphane (for ALDH3A1) [25,42,43]. It should be noted that the RALDHs/ALDH2 activities were measured by monitoring the generation of NADH from NAD^+^, coupled with aldehyde dehydrogenation. While star anise, cumin seed and caraway seed all contain various aldehyde compounds, several phyto-derived aldehydes have been reported to be favorably oxidized by RALDH1 [34]. Therefore, it is possible that the increase in NADH production by RALDH1 is due to the oxidation of alternative substrate(s) and it is necessary to further investigate whether these herbs and spices contain novel activators for RALDH1. Since little is known about the effects of spices/herbs on ALDHs including RALDHs *in vitro*, the results reported here may contribute to further studies on natural phyto-derived modulators for this enzyme family, which may contribute to not only basic research but also practical purposes including drug and functional food development.

### The major component in star anise extract shows no inhibitory effect for RALDHs/ALDH2

**Figure 5:**
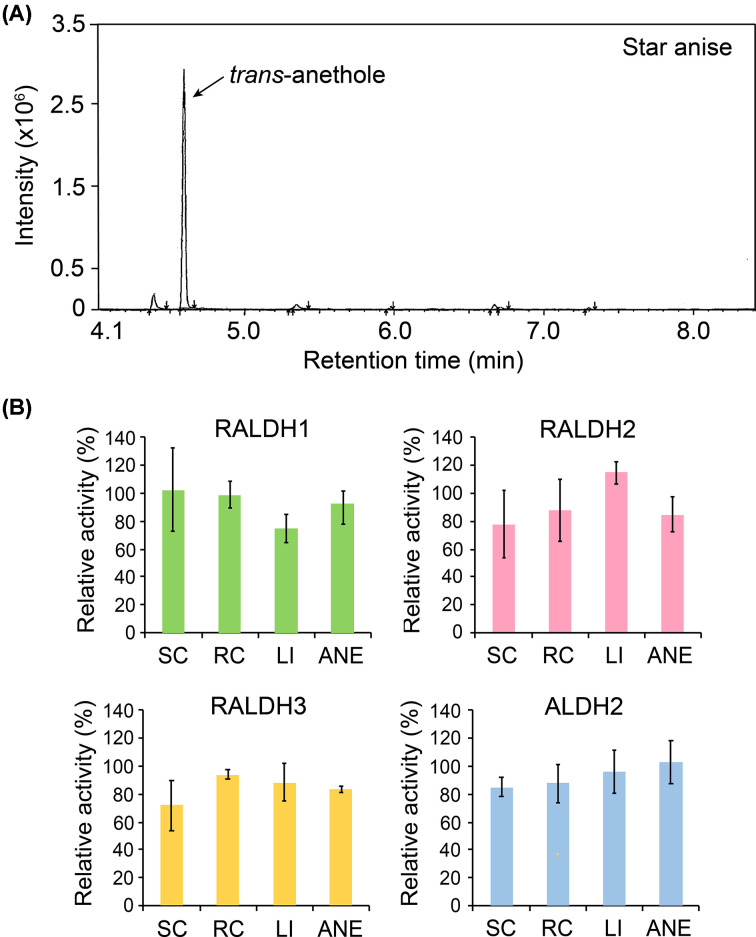
The major component ANE in the star anise extract show no inhibitory effect for RALDHs/ALDH2 (**A**) Total ion chromatograms of the star anise extract with its most abundant component ANE. (**B**) Effects of monoterpenes including ANE on the activity of RALDHs/ALDH2. SC: *S*-(+)-carvone, RC: *R*-(−)-carvone. Error bars indicate standard deviations of mean at 95% confidence (*n*=3).

For confirmation of the most abundant components in star anise which showed specific inhibitory effect for RALDH3 (Figure 4B–E), we performed gas chromatography/mass spectrometry (GC/MS) analysis on the extract. Our total ion chromatogram showed a highly similar composition result as previously reported, with the monoterpene *trans*-anethole (ANE) accounting for over 70% (Figure 5A) [44]. ANE was subjected to the *in vitro* activity assay of RADLHs/ALDH2. For comparison, we also tested three other monoterpenes including LI, *S*-(+)-carvone and its isomer *R*-(-)-carvone that are reported to be the major components in many essential oils and extracts from herbs and spices [45]. However, ANE, as well as the other three monoterpenes, did not exhibit any significant effects on the activities of RALDHs/ALDH2 unlike the extract of star anise (Figure 5B). Three extracts of star anise, fennel seed and anise seed showed different selective effects for RALDHs/ALDH2 (Figure 4B–E) despite sharing the same most abundant compound as reported in previous studies as well as confirmed with GC/MS [46,47] (Figure 5A and Supplementary Figure S5). Therefore, these effects can be attributed to the different compounds that are present in trace amounts in individual spices.

For each plant, only one or a few bioactive compounds are present in abundant amounts while the number of trace compounds in plants can be enormous, which burdens the investigation of target bioactive components. Hence, our results suggest that there is a high possibility that the specific inhibitors for RALDHs in the found extracts may be present in trace amounts with very potent activity or compounds that are considered minor. Although the RALDHs are known to enhance tumor activity, one of the isoforms, RALDH2, exhibits important roles in immune responses [48,49]. Therefore, these selective inhibitors are required to preserve functions of such beneficial isoforms and the subsequent identification of their chemical structures is of critical importance.

## Conclusion

In the present study, we proposed a simplified and unified method to prepare recombinant proteins of three human RALDH isozymes in the common way and to measure their activity using the natural substrate atRAL. Appropriate deletion of the N-terminal disordered region facilitated simple expression of RALDHs/ALDH proteins using the universal pET-vector and *E. coli* expression system. In addition, magnesium ion was observed to have differential effects on atRAL dehydrogenation activity of RALDHs, which is consistent with previous studies using alternative substrates like hexanal. The established method for measuring RAL dehydrogenation activity in this study will accelerate future research to dissect molecular mechanisms of the metal-depending regulation and the substrate specificity of all the three human RALDHs. Furthermore, we demonstrated that N-terminal His-tag does not attenuate the activity of RALDHs with N-terminal disordered region deleted.

To find phyto-derived selective modulators for RALDHs, we prepared ethanol extracts of herbs and spices, which are recognized as functional foods and repository of phytochemicals. The RALDHs inhibitory assays were performed using the whole extracts rather than a single identified compound, with an expectation that spices and herbs would contain unidentified functional trace components. A potential presence of selective potent inhibitor(s) was shown in star anise for RALDH3, but not for RALDH1/2. Moreover, we showed that the common compound that is abundant in star anise, ANE, does not inhibit RALDH3. These results imply that the target compound(s), which may be the strong and selective inhibitor(s) for RALDH3, exist in only trace amounts in star anise.

Altogether, we propose a promising workflow to find selective modulators for RALDHs and suggest potential sources of selective modulators which are derived from medicinal plants including herbs and spices. Furthermore, the current study highlights the importance of further focus on trace phytochemicals in regulation of RALDHs and RA-governed metabolic pathways in particular as well as immunotherapy and cancer therapy in general.

## Materials and methods

### Chemicals

Most chemicals were provided by FUJIFILM Wako Pure Chemical Corporation (Osaka, Japan) with the following exceptions: human liver/brain total RNA from TaKaRa Bio Inc. (Shiga, Japan), Triton X-100 from Anatrace Products LLC. (Perrysburg, U.S.A.), and protease inhibitor cocktail from Nacalai Tesque (Kyoto, Japan). Recombinant untagged HRV 3C protease was prepared by *E. coli* expression system and two-step purification using anion- and cation-exchange chromatography.

### Phylogenetic analysis of the human ALDH genes

Phylogenetic analyses were conducted in Mega X [50] using the canonical nucleotide sequences of 19 human *ALDH* genes. The evolutionary history was inferred using the Neighbor-Joining method [51]. The optimal tree with the sum of branch length = 8.99577651 is shown. The percentage of replicate trees in which the associated taxa clustered together in the bootstrap test (500 replicates) are shown next to the branches [52]. The tree is drawn to scale, with branch lengths in the same units as those of the evolutionary distances used to infer the phylogenetic tree. The evolutionary distances were computed using the Maximum Composite Likelihood method [53] and are in the units of the number of base substitutions per site. Codon positions included were 1st+2nd+3rd+Noncoding. All ambiguous positions were removed for each sequence pair (pairwise deletion option). There was a total of 4316 positions in the final dataset.

### *E. coli* expression of RALDHs/ALDH2 recombinant proteins

Full-length cDNAs of human *RALDH1* (*ALDH1A1*), *RALDH2* (*ALDH1A2*), *RALDH3* (*ALDH1A3*) and *ALDH2* (NCBI, accession numbers NM_000689.5, NM_003888.4, NM_001293815.2 and NM_000690.4, respectively) were synthesized by reverse-transcriptase PCR from Human Liver/Brain Total RNA (TAKARA). The disordered regions for deletion within the four human RALDHs/ALDH2 were predicted by XTalPred-RF server with reference to previous studies. Truncated stretches coding for functional human RALDH1 (Leu^9^–Ser^501^), RALDH2 (Leu^26^–Ser^518^), RALDH3 (Leu^20^–Pro^512^) and ALDH2 (Ser^18^–Ser^517^) were BamHI-introduced into pET-47b(+) (Novagen) and cloned to Rosetta(DE3) (Novagen). Overexpression was induced by 0.5 mM isopropyl-β-d-(-)-thiogalactopyranoside (IPTG) at 18°C for RALDH1-3 and at 16°C for ALDH2. After incubation for 18 h, cells were harvested by centrifugation at 5000 rpm for 10 min and stored at −80°C until use. Harvested cells were resuspended in buffer A (50 mM 4-(2-hydroxyethyl)-1-piperazineethanesulfonic acid (HEPES) pH 7.5, 300 mM NaCl, 10 mM imidazole, 0.5 mg/ml lysozyme and 0.01% Triton X-100) with addition of 0.1% (v/v) protease inhibitor cocktail (Nacalai Tesque). The resuspended cells were lysed by sonication with SONIFIER Model 250D (BRANSON). Cell debris was thoroughly removed by centrifugation at 40000×***g*** for 30 min at 4°C. Crude protein fractions were applied to Ni-NTA superflow resin (Qiagen) and were either eluted with buffer B (50 mM HEPES pH 7.5, 300 mM NaCl and 200 mM imidazole) to obtain His-tagged RALDHs/ALDH2 proteins, or incubated with HRV 3C protease overnight to remove His-tag before being eluted with buffer C (50 mM HEPES pH 7.5, 300 mM NaCl and 10 mM imidazole) to obtain untagged RALDHs/ALDH2 proteins. Finally, all proteins were further purified by SEC using a Superdex 200 10/300 GL column (GE Healthcare) in an ÄKTA pure protein purification system (GE Healthcare). SEC was performed using buffer E (50 mM HEPES pH 8.0 and 150 mM KCl) at 0.3 ml/min flow rate and 4°C. Purified proteins were measured for concentration by their absorbance at 280 nm using NanoDrop ND-1000 (Thermo Fisher Scientific) and visualized by SDS/PAGE.

### Determination of enzyme activity of RALDHs/ALDH2

Enzyme activity of RALDHs/ALDH2 was indirectly determined via distinct fluorescence signal of NADH converted from NAD^+^ upon oxidation of the natural substrate atRAL into all-*trans*-RA (atRA) at excitation and emission wavelengths of 340 and 470 nm, respectively. atRAL was freshly prepared on the day of an assay with concentration determined by absorption spectroscopy as previously described [54]. For determination of the effects of magnesium ions and His-tag on the activity of RALDHs/ALDH2, assays with 20 μl of 4.6 μM His-tagged or untagged enzyme in buffer E (final concentration: 460 nM), 20 μl of 1 mM NAD^+^ (final concentration: 100 μM) and initiated by the addition of 20 μl of 120 μM atRAL substrate dissolved in ethanol (final concentration: 12 μM) were separately conducted in three HEPES-based buffers (total volume: 200 μl) with and without magnesium as follows: RB1 (20 mM HEPES pH 8.5, 150 mM KCl, 1 mM ethylenediaminetetraacetic acid (EDTA) and 20% 2-methyl-2,4-pentanediol (MPD) [20]), RB2 (50 mM HEPES pH 8.0, 50 mM KCl, 0.5 mM EDTA, 0.5 mM dithiothreitol (DTT) and 5% MPD) and RB3 (50 mM HEPES pH 8.0, 50 mM KCl, 0.5 mM EDTA, 0.5 mM DTT, 5% MPD and 30 mM MgCl_2_ [55]). For determination of the effects of various spice and herb extracts on RALDHs/ALDH2, assays with 20 μl of 4.0 μM untagged enzyme in buffer E (final concentration: 400 nM), 20 μl of 1 mM NAD^+^ (final concentration: 100 μM), 20 μl of extract (final concentration: 5% of reaction mixture) in ethanol 60% and initiated by the addition of 20 μl of 120 μM atRAL substrate dissolved in ethanol (final concentration: 12 μM) were conducted in RB1 for RALDH1/2 and ALDH2 or buffer RB3 for RALDH3 (total volume: 200 μl). The change in NADH fluorescence signals was monitored by SpectraMax iD5 multimode microplate reader (Molecular Devices) in 96-well polystyrene black FLUOTRAC microplate (Greiner bio-one) for 5 min with 30-s intervals. Initial velocity *V*_0_ of reactions were calculated as the slope of the initial linear portion of the reaction progress curve [56]. Relative activity of enzyme under treatment was expressed by percentage of control (normalized to 100%) of each run. All assays were conducted for at least three replications.

### Extraction of spice and herbs

Twenty-two kinds of dry spices and herbs including juniper berry (*Juniperus* sp.), star anise (*Illicium verum*), mace (*Myristica fragrans*), shallot (*Allium cepa* var. aggregatum), chive (*Allium schoenoprasum*), turmeric (*Curcuma longa*), chervil (*Anthriscus cerefolium*), cumin seed (*Cuminum cyminum*), dill (*Anethum graveolens*) seed and weed, celery seed (*Apium graveolens*), fennel seed (*Foeniculum vulgare*), caraway seed (*Carum carvi*), anise seed (*Pimpinella anisum*), marjoram (*Origanum majorana*), oregano (*Origanum vulgare* L.), rosemary (*Rosmarinus officinalis*), sage (*Salvia officinalis* L.), thyme (*Thymus vulgaris* L.), cinnamon (*Cinnamomum verum*), laurel (*Laurus nobilis* L.), clove (*Syzygium aromaticum*) and horseradish (*Armoracia rusticana*) were purchased from S&B Foods Inc. Dry spices and herbs were soaked in ethanol 60% in airtight capped tubes at ratio of sample: solvent of 1:10 (w/v). Tubes were subjected to continuous mixing for 48 h at room temperature, protected from light. Clear extracts were collected by centrifugation while residues were extracted for two more times using the same protocol and new solvent to maximize extraction efficiency. Extracts of three-time extraction were combined, filtered through 0.45-μm Millipore Express Polyethersulfone (PES) Membrane (Merck Millipore, Ltd.) and stored at 4°C protected from light until use.

###  GC/MS analysis

GC/MS was performed on a Shimadzu GCMS-QP2010 Plus system equipped with an AOC20i+s autosampler and a Rtx-5MS fused silica 30 m × 0.25 mm × 0.25 μm column (Restek Corp.). Helium was used as the carrier gas at 100 kPa. Samples were injected at a split ratio of 1:10 and ionization voltage of 70 eV. Temperatures of the column oven and GC/MS interface was 100 and 250°C respectively. Column temperature was started at 100°C, held for 1 min, then ramped at 20°C/min to 300°C, held for 5 min before decreasing to 100°C at 40°C/min. Data were acquired and analyzed using GCSolution software (Shimadzu). Scanning was performed over a mass range of 40–550 amu and constituents were identified by comparison with National Institute of Standards and Technology (NIST) libraries and literature data.

### Statistics

Mean values and standard deviations were calculated using Microsoft Excel 2019 (Microsoft Corp., San Leonardo, CA, U.S.A.). Statistical significance was analyzed by one-way analysis of variance (ANOVA) with Tukey’s *post hoc* tests using SPSS 26 (IBM Corp.).

## Supplementary Material

Supplementary Figures S1-S5

## Data Availability

The datasets generated during and/or analyzed during the current study are available from the corresponding authors on reasonable request.

## Open Access

Open access for this article was enabled by the participation of University of Tokyo in an all-inclusive *Read & Publish* pilot with Portland Press and the Biochemical Society under a transformative agreement with USACO.

## Abbreviations

ALDH: aldehyde dehydrogenase
ANE: *trans*-anethole
atRA: all-*trans*-retinoic acid
atRAL: all-*trans*-retinal
CSC: cancer stem-like cell
DTT: dithiothreitol
EDTA: ethylenediaminetetraacetic acid
GC/MS: gas chromatography/mass spectrometry
HEPES: 4-(2-hydroxyethyl)-1-piperazineethanesulfonic acid
HRV 3C: human rhinovirus 3C
LI: limonene
NAD^+^/NADH: nicotinamide adenine dinucleotide
RA: retinoic acid
RAL: retinaldehyde (retinal)
RALDH: retinaldehyde dehydrogenase
RB: reaction buffer
SDS/PAGE: sodium dodecyl sulfate/polyacrylamide gel electrophoresis
SEC: size-exclusion chromatography
