# Supplementary material for: Evaluation of spice and herb as phyto-derived selective modulators of human retinaldehyde dehydrogenases using a simple in vitro method
Source: Biosci Rep. 2021 May 20;41(5):BSR20210491. doi: 10.1042/BSR20210491 (PMC8493444; doi:10.1042/BSR20210491)
Supplement: Supplementary Figures S1-S5 [file BSR-2021-0491_supp.pdf]

Supplementary Material

Evaluation of spice and herb as phyto-derived selective modulators for human retinaldehyde dehydrogenases using a simple *in vitro* method

Thi Bao Chau Bui[a,b,c], Shohei Nosaki[a,d], Mito Kokawa[d], Yuqun Xu[a], Yutaka Kitamura[d], Masaru Tanokura[a,b,*], Satoshi Hachimura[a,b,*], Takuya Miyakawa[a,*]

[a] *Department of Applied Biological Chemistry, Graduate School of Agricultural and Life Sciences, The University of Tokyo, Tokyo 113-8657, Japan*

[b] *Research Center for Food Safety, Graduate School of Agricultural and Life Sciences, The University of Tokyo, Bunkyo-ku, Tokyo 113-8657, Japan*

[c] *Graduate School of Science and Technology, University of Tsukuba, 1-1-1 Tennodai, Tsukuba, Ibaraki 305-8572, Japan*

[d] *Faculty of Life and Environmental Sciences, University of Tsukuba, 1-1-1 Tennodai, Tsukuba, Ibaraki 305-8572, Japan*

* Corresponding authors.

E-mail address: amtanok@mail.ecc.u-tokyo.ac.jp (M. Tanokura), ahachi@g.ecc.u-tokyo.ac.jp (S. Hachimura), atmiya@mail.ecc.u-tokyo.ac.jp (T. Miyakawa).

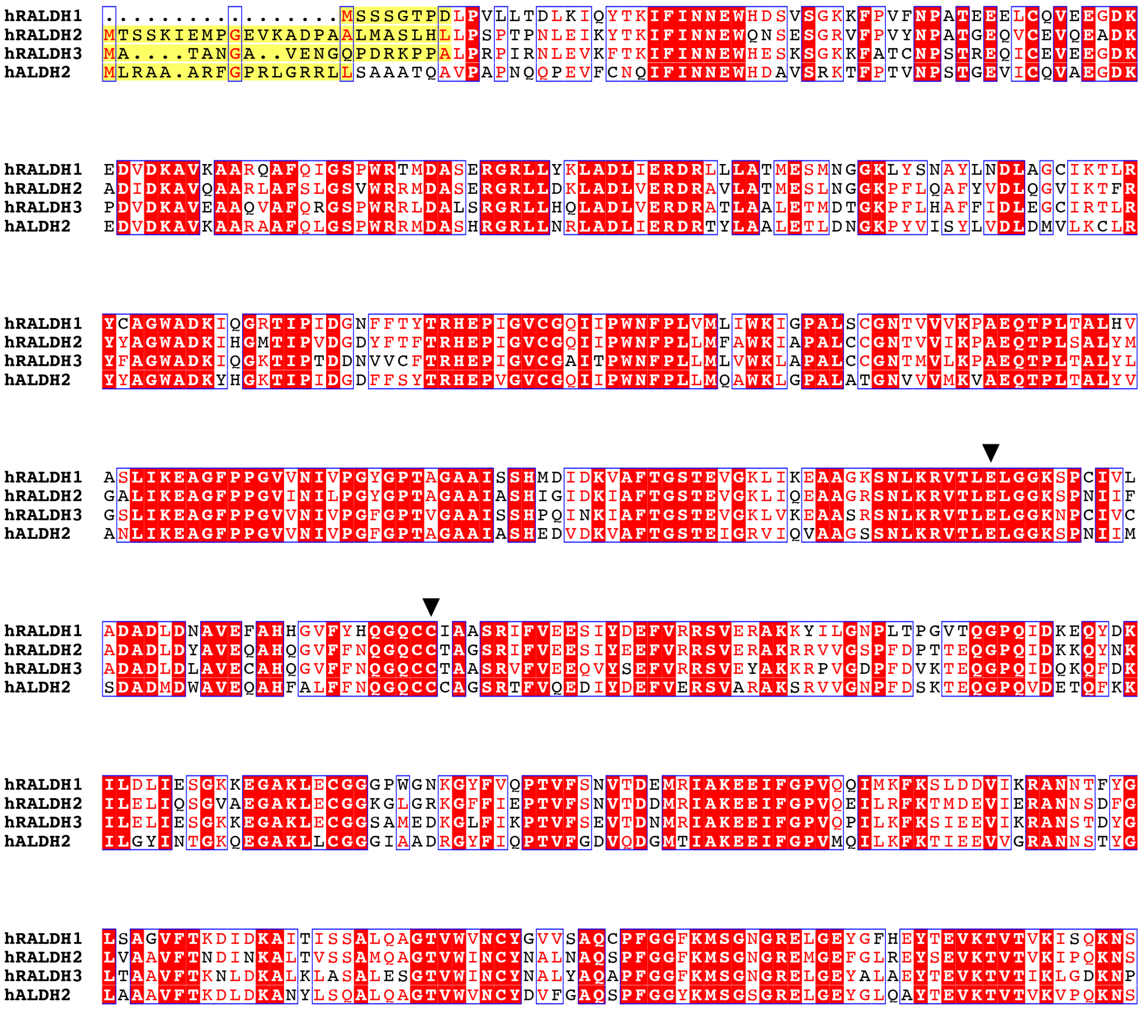

Figure S1. Multiple sequence alignment of human RALDHs and ALDH2. Identical residues are shaded red, and homologous residues are shown as red letters. Alignment of sequences were performed by CLUSTAL W and displayed by ESPript 3.0. Arrowheads indicate conserved catalytic residues Cys and Glu. Yellow highlights indicate N-terminus deleted regions.

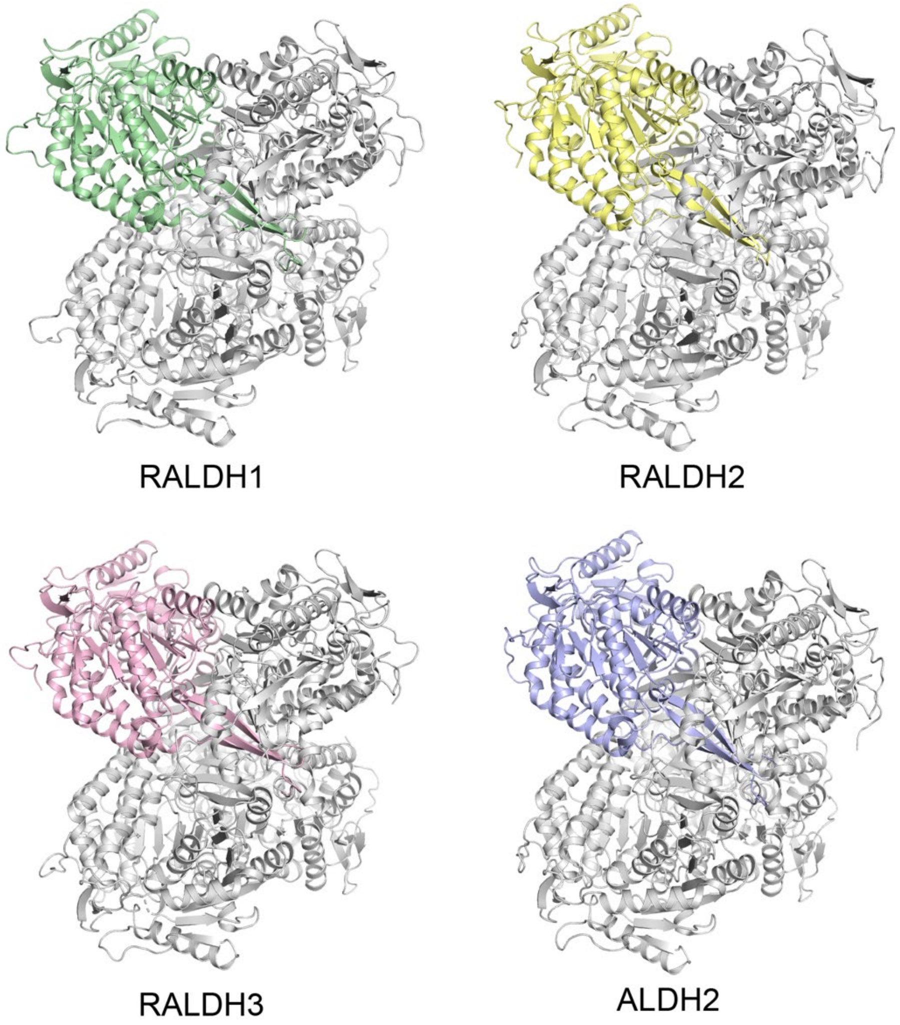

Figure S2. RALDHs and ALDH2 share highly homologous structures. Tetrameric structures of RALDH2, RALDH1, RALDH3 and ALDH2 were created based on Protein Data Bank (PDB) code 1BI9, 4WB9, 5FHZ and 1BI9, respectively. One protomer of each tetramer is colored.

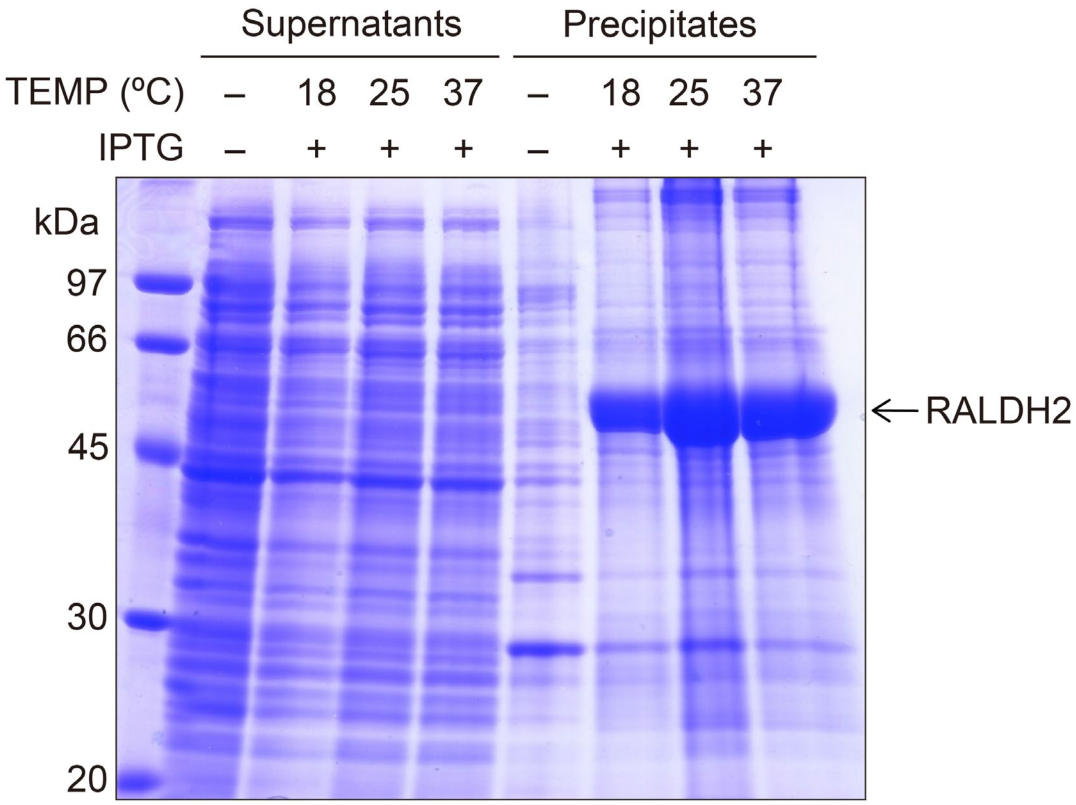

Figure S3. Coomassie-stained SDS-PAGE analysis for *E. coli* expression of the full-length RALDH2. The expression was conducted using plasmid pET-47b(+) and host strain Rosetta(DE3) with 0.5 mM IPTG induction at 18 ℃ overnight, at 25 ℃ overnight or at 37 ℃ for 2 h.

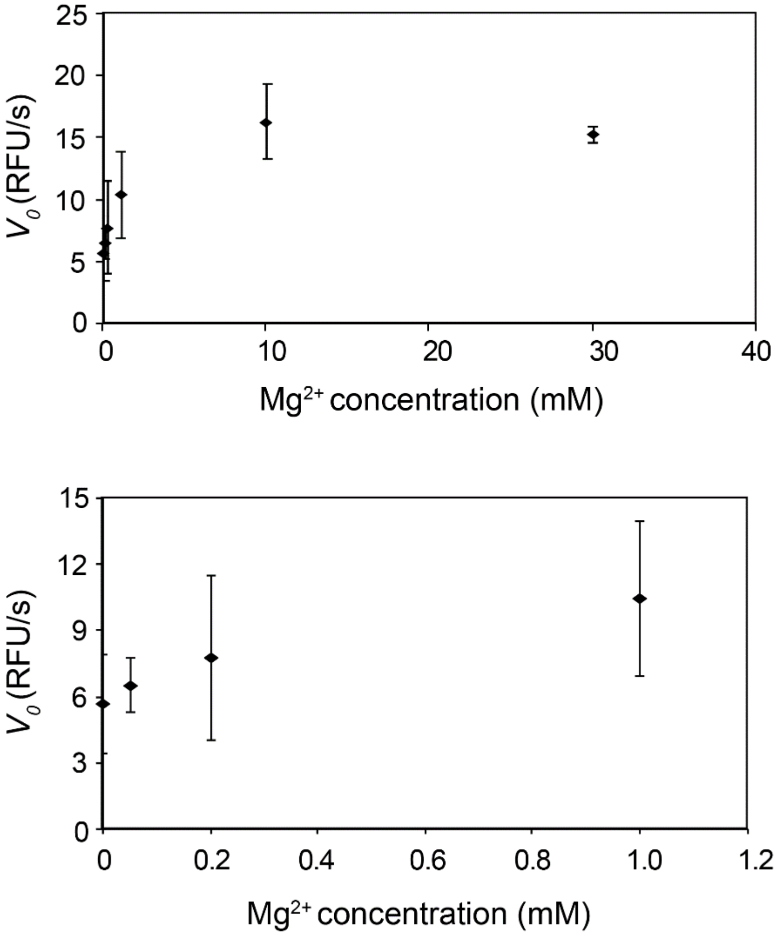

Figure S4. Effects of Mg^{2+} ion concentration on the RAL dehydrogenation reactivity of RALDH3. Upper and lower panels show Mg^{2+} ion concentration ranges of 0−30 mM and 0−1.0 mM, respectively. Error bars indicate standard deviations of means (n = 3).

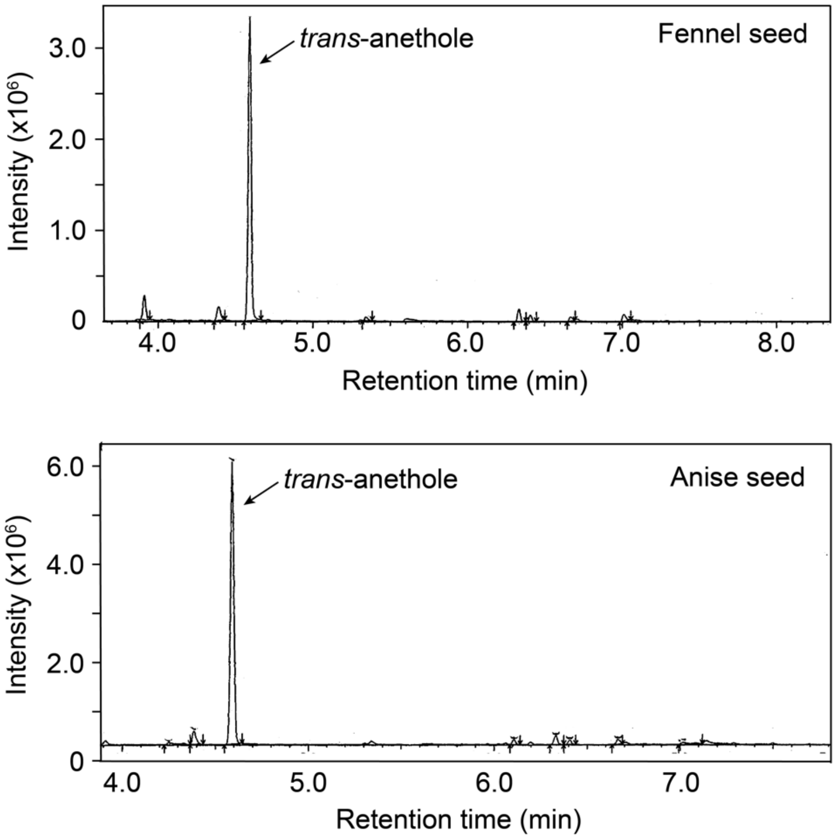

Figure S5. Total ion chromatograms of the fennel seed (top) and the anise seed (bottom) extracts with their most abundant component *trans*-anethole.
